# Optimizing the Explosive Force of the Elite Level Football-Tennis Players through Plyometric and Specific Exercises

**DOI:** 10.3390/ijerph18158228

**Published:** 2021-08-03

**Authors:** Anamaria Gherghel, Dana Badau, Adela Badau, Liviu Moraru, Gabriel Marian Manolache, Bogdan Marian Oancea, Corina Tifrea, Virgil Tudor, Raluca Maria Costache

**Affiliations:** 1 Faculty of Physical Education and Sports, National University of Physical Education and Sports, 060057 Bucharest, Romania; anemaria.gherghel@yahoo.com (A.G.); c_tifrea@yahoo.com (C.T.); virgiltro@yahoo.com (V.T.); rally_kyn@yahoo.com (R.M.C.); 2 Faculty of Sciences and Letters, “George Emil Palade” University of Medicine, Pharmacy, Sciences and Technology, 540142 Targu Mures, Romania; adela.badau@umfst.ro; 3 Faculty of Physical Education and Mountain Sports, Transilvania University, 500068 Brasov, Romania; bogdan.oancea@unitbv.ro; 4 Faculty of Medicine, “George Emil Palade” University of Medicine, Pharmacy, Sciences and Technology, 540142 Targu Mures, Romania; 5 Faculty of Physcial Education and Sports, “Dunarea de Jos” University, 800003 Galati, Romania; gabriel.manolache@ugal.ro

**Keywords:** football-tennis, explosive force, fitness-physical, physical performance, contact time, flight time, bounce height, bounce strength, Reactive Strength Index

## Abstract

The aim of the research was to implement an athletic program to improve the explosive force in order to optimize physical fitness at the level of elite football-tennis players and evaluate the progress made through specific tests using the Opto Jump. The research included 10 elite European and world-class players, on whom an experimental program was applied in order to improve the explosive force of the limbs in conditions of speed, endurance, and dynamic balance. Study tests: five vertical jumps on the spot, on the left/right leg; five back and forth jumps on the left/right leg; five left/right side jumps on the left/right leg; vertical jumps on both legs 60 s; BFS vertical jumps. For each test, the following parameters specific to the explosive force were statistically analyzed: contact time (s); flight time (s); jump height (cm), jump power (w/kg); RSI—Reactive Strength Index, defined as Height (m/s). In the study, the average value of the parameters specific to the jumps performed in each test was taken into account. During the study, the tests were performed and processed on the Opto Jump device and software. In all tests of the experiment monitored through Opto Jump, significant progress was made in the final test compared to the initial one, which demonstrates the efficiency of the physical training program implemented for the development of explosive force, with an impact on the sports performance of elite players. The most relevant results obtained for the left leg regarding the improvement of the explosive force of the lower limbs materialized in the jump height parameter was in the test of five vertical jumps on one leg on the spot, and for the right leg in the tests of: five back and forth jumps and five left/right side jumps. The most significant advances in the study were in the tests, in descending order of their weight: 60 s vertical jumps on both legs; five back-and-forth jumps and five left/right side jumps, five vertical jumps on one leg standing, and BFS vertical jumps.

## 1. Introduction

The game of football-tennis is a relatively new sport, which is in a continuous dynamic, being able to be practiced both as a performance, competitive sport, and as a recreational activity. Football-tennis is the result of combining football skills with the rules of tennis and volleyball, which can be practiced indoors and outdoors. The main skills specific to football-tennis are: passing the ball, passing the ball over the net, service; technical skills with various parameters of direction, speed and strength in order to award the point and implicitly the victory [1]. The football-tennis game demands from the players a superior level of physical fitness and an advanced playing technique.

The elite sports performance in the football-tennis game targets complex athletes, with a very good level of technical skills under the following conditions of execution: speed, coordination, explosive force, and endurance. The high performance football-tennis game has various forms of organization and can be practiced as: single game (individual), doubles game, triple game, mixed doubles game, and mixed triple game. Soccer-tennis players require specialized physical training embodied in dynamic muscular performance to perform technical skills, changes of direction, vertical and horizontal jumps in resistance and speed, as well as power and accuracy for hitting the ball [2]. Football-tennis at the competitive level requires athletes to activate the main muscle groups with a focus on the muscles of the lower limbs, an optimal functioning of the cardio-respiratory system [3,4,5,6], a proper adaptation of the vestibular apparatus [7,8,9,10], and metabolic processes [11,12]. The benefits of playing football-tennis are multiple, having an impact on physical capacity, mental capacity, communication and social integration capacity, team spirit formation, etc. Football-tennis trainings must focus on improving physical fitness, on improving the control of movements in ball handling, on improving the general strength, the explosive force, coordination, dynamic balance, reaction capacity, kinesthesia, accuracy movements, and spatial orientation. Football-tennis is a dynamic activity, with variable effort intensity, from maximum to moderate depending on the phases of the game. Muscle training specific to strength development requires training adapted programs, typical of the game of football-tennis that must be based on in-depth knowledge of the skeletal muscle structure of the lower limbs (gluteal muscles, thigh muscles, tailor, femoral quadriceps, femoral biceps, thigh adductors), leg muscles (gastrocnemius, pronators and supinator of the leg, flexors and extensors), and plantar muscles (extensors of the toes and soles)), the type of voluntary muscle contractions, how to select and activate muscular fibres, and complex mechanisms specific to muscle contraction [11,12,13,14,15]. The balanced development of the muscles of the lower limbs of the football-tennis players represents an important condition for the optimization of the technical and physical performances [16,17,18,19].

The human ability to develop muscle strength in the shortest possible time is called explosive force, which is conditioned by: age, level of sports training, the volume and intensity of training, and the nature of the training stimulus [13]. Studies have shown the impact of high levels of the explosive force at the level of the lower limbs on the improvement of the pass, of the kicks from the ground, of the blows from the jump, of the changes of direction, of the slits, etc. [20,21,22]. The explosive force is variable depending on the individual particularities [23], especially in the first phase (the first 50 m/s) [24], as well as on the muscular architecture [25,26]. The explosive force is conditioned by the speed of the electrical signals along the surface of the muscle fibers and the dominant muscle fiber type, white fibers (type IIb), facilitating rapid contraction [27,28,29]. The explosive force of the lower limbs is dependent on the size of the motor units that stimulate muscle contraction, metabolic processes, capillary density (the amount of blood that reaches them), and mitochondrial density [30,31]. A peculiarity of physical training is related to the functional specialization of the human body that leads to the demand for strength and endurance with the peripheral neuromuscular apparatus, through muscle hypertrophy, the ratio of intra and intermuscular coordination, and increased metabolism [32,33,34,35]. The increase of the absolute force, of the mechanical force related to the explosive effort is achieved by increasing the energetic potential of the muscles [36,37].

The analysis of the literature highlights that there is currently not much existing research on football-tennis. Additionally, important components of sports training such as the interactions between anthropometric parameters, biological maturation, and physical performance, and the level of specific technical skills, are insufficiently and unidirectionally addressed [13,38]. Jumping skills conditioned by explosive force of the upper limbs is approached in many sports games, such as football [39,40,41], handball [42,43], volleyball [44,45,46], basketball [47,48], and other, but very few in football-tennis [49,50]. The explosive force of the lower limbs represents an essential component of physical fitness that conditions the sports performances in football-tennis, at the level of all training levels, but relevantly at the level of the elite athletes who participate in top sports competitions.

The aim of the research was to implement an athletic program to improve the explosive force in order to optimize physical fitness at the level of elite football players and evaluate the progress made through specific tests using the Opto Jump. The novelty of our study is the review of the athletic exercise program applied to a sample of elite athletes in football-tennis and in the evaluation of various tests specific to the explosive force in speed, endurance, and dynamic balance through the Opto Jump. The originality of the research topic is represented by the fact that the research is carried out on athletes from the Romanian national team of football-tennis. We consider this a major impact on this sport, which is not very developed and known in Romania, but has major and notable international results that dominate the world and European top from 2010 to the present. It is also the only research at a national level carried out on football-tennis team athletes in an organized and centralized setting.

## 2. Materials and Methods

### 2.1. Study Design and Practical Implications

The research took place at the Romanian Olympic and Sports Complex—Izvorani. The initial testing was performed between January 7 and 15, 2019, and the implementation of the program occurred from 16 January to 18 November 2019. The final testing was performed between November 20 and 28, 2019. The physical tests applied to the experimental sample aimed at the explosive force at the level of the lower limbs, in conditions of speed, resistance, and dynamic balance at the level of the lower limbs, the tests were: five vertical jumps on the spot, on the left/right leg; five back and forth jumps on the left/right leg; five left/right side jumps on the left/right leg; vertical jumps on both legs 60 s; and BFS vertical jumps. For each test, the following parameters specific to the explosive force were statistically analyzed: contact time (s); flight time (s); jump height (cm), jump power (w/kg); and RSI—Reactive Strength Index, defined as Height (m/s). For the current study, the maximum value achieved at the best jump from the five jumps included in the standardized test that evaluates the strength in endurance regime was taken into account.

In the study, the average value of the parameters specific to the jumps performed in each test was taken into account. During the study, the tests were performed and processed on the Opto Jump device and software. Opto Jump consists of an optical measurement system that consists of a transmission and reception bar, with dedicated software that facilitates the recording of physical fitness parameters associated with the athlete’s performance with maximum accuracy and in real time [51]. In this study we opted for Opto Jump, which is a mobile platform that was used both in the process of assessing physical capacity and in the players’ program due to the possibility of using it in the training room and in the testing laboratory. In sports practice, a series of famous sports teams and institutions use the Opto Jump platform in the training and testing process, among the following teams: Juventus, AC Milan, AS Roma, Real Madrid, Standard Liege, as well as the Olympic Center in California and Colorado SU; Italian Tennis Federation, German Tennis Federation, National Athletics Federations from: Spain, Germany, Great Britain.

The study was conducted according to the guidelines of the Declaration of Helsinki and was approved by the Institutional Review Board of National University of Physical Education and Sports, Bucharest, Romania, protocol no. 4271, with the date of approval being the 31 October, 2017. For this article, all authors contributed equally; all authors have an equal contribution to the publication with the first author, too.

The program for the development of physical fitness focusing on explosive force was implemented over a period of three months, comprising between five and nine workouts per week, with an average duration of 90 min. During the basic training period (six weeks) the structuring of the trainings was 70% physical training and 30% technical training, and in the pre-competition period (six weeks) it was 60% physical training and 40% technical training. The exercises were adapted to the specifics of the football-tennis game. On average, the workload was 90%, with an intensity between 60 and 70%. The exercises included in the training program for improving the explosive force included: plyometric exercises, jumping exercises, technical exercises with acceptance on hanging on one leg or both legs, passes without jumping, hitting the ball from the jump, etc. The program was individualized according to the sports peculiarities of the subjects included in the study.

Each of the 10 athletes included in the study represents a case study, because they have outstanding performances in the world and are among the European elite in singles, doubles, mixed doubles, triples, and mixed doubles tennis in the last 10 years. The components of the study represent the most valuable Romanian football-tennis athletes who are medaled worldwide and in Europe. The practical implications of this study aim to identify the impact that physical training programs that include plyometric exercises and technical exercises adapted to improve lower limb strength will facilitate the optimization of the physical performance level of football-tennis athletes. The training program implemented and adapted to the individual motor and technical particularities of elite athletes will be able to be examples of good practices for the physical training of football-tennis players. Adapting and applying the tests included in the current study to the technical and performance requirements of the football-tennis game can become effective tools for testing the physical capacity in general and the strength of the lower limbs in particular.

Using the means and methods of playing tennis and finding a balance between the multilateral and specialized training (with the ball) of each player, the aim was to mainly develop skills, and especially strength. Personalized training is done through specific efforts that simulate or reproduce game situations, targeting certain biomotor qualities of the player such as strength, speed, endurance, coordination skills, and combined. The strong development, from a specific point of view, was achieved through competition ball exercises adapted to the technical specifics of the football-tennis game. The following are a few examples of the mentioned ball exercises: the performer is positioned on the left side line of the playing field and 2 m from the net—lying face down with palm support, performing flexion and extension in the elbow joint with a vertical jump—self-pass and long diagonal attack with the soccer-tennis ball (3 × 10 × 20 × 30”px70%), volume 90% and intensity 70%; dosage 1–3x; break 1 min passive; line-of-work formation; sitting at 4 m from the net, the ball to the player—taking the ball wide and throwing the ball over his head—turning—taking—attacking from above in any part of the field of play. This is executed without letting the ball fall to the ground (3 × 5 × 10 × 30”px80%), volume 90% and intensity 70%: dosage 1–3x; passive 30 s pause; work formation—line in a row; sitting with the ball—the executor is at the 9 m line of the football-tennis court and has to execute the takeover followed by throwing the ball to the 6.40 m line, running; taking the ball without touching the ground—throwing the ball over his head at the 9 m line—running—taking the ball—long line attack (3 × 20 × 30”px70%), volume 90% and intensity 70%; dosage 1–3x; passive 30 s pause; work formation—line in a row.

### 2.2. Subjects

The experimental sample included 10 senior, female performance athletes, multiple world and European football-tennis champions, mean age ± SD 2.56 ± 056 years; height 166.01 ± 5.37 cm; weight 62.10 ± 6.48 kg; BMI 22.52 ± 1.96. Inclusion criteria: active sports, age over 18 years, good health, no injuries in the last 3 months, components of national teams, and European or world champions.

### 2.3. Statistical Analyzes

The results of the study were processed using SPPS 24 software, the main statistically calculated indicators were: arithmetic mean (X), standard deviation (SD), Student test (t), average differences between the final test and the initial test (∆XTI-TF), interval of confidence (95% CI lower; upper), percentage of progress (PG%), and Cohen’s (d) for effect size. Interpretation of effect size: small (0.2), medium (0.5) and large (0.8) [52]. Statistical significance was set at *p* < 0.05 for all analysis.

## 3. Results

The statistical processing of the test allows for the analysis of the differences of the arithmetic means, thus the executions on the right leg are better than for the left leg for the parameters: contact time −0.004, flight time 0.019; while the executions on the left leg were better than on the right leg at: jump height 0.92, jump strength 0.83, RSI 0.06. The processing of the results (Table 1) for the test of five vertical jumps on the spot between the executions on the left leg compared to the right leg highlights the following differences: contact time −0.001, flight time −11, jump height −1.54, jump strength −1.79, RSI 0.08; and at the final test: contact time −0.001, flight time −0.001, jump height −0.62, jump power −0.96, RSI −0.03. At all test parameters the results in the final test were better than in the final test, the highest progress was registered at RSI 113.1% for the left foot and 69.3% for the right foot. Most of the parameters analyzed in this test recorded a large effect size over 0.08, except for execution on the left leg at the contact time parameter where the effect size was 0.46 on average. All parameters analyzed recorded statistically significant results, except for the contact time on the left leg, which was *p* = 0.178.

The Table 2 analysis highlights that all results were statistically significant at all test parameters. The highest progress registered at the execution on the standing leg was at the parameters RSI 247.1% and the jump height 116.9%, and for the executions on the right leg at RSI 184.3% and the flight time 135.1%. Comparing the average of the differences registered between the two tests and between the executions of the standing and right leg, the following relevant results are highlighted: the contact time was better on the right leg with 0.234, the flight time was better on the right leg with 0.126, the height the jump was better for the right foot with 0.141, the jump power was equal for the jumps on the left and right foot; and RSI was better on the left leg with 0.003. At all test parameters, for the executions on both legs, the effect scale was medium and wide.

From Table 3, the results of the test of five left/right side jumps on the left/right leg facilitated the statistical analysis of the differences of the arithmetic means at the initial test, so the executions on the left leg were better than for the right leg for the parameters: time contact 0.107; jump power 1.14, RSI 0.04; executions on the right leg were better than those on the left leg in time flight -.022 and jump height 0.047. Processing the results (Table 3) in the test of five vertical jumps on the spot statistically analyses the differences of the arithmetic means at the final test, so the executions on the right leg were better than those on the left leg at all parameters: contact time 0.016, flight time 0.011, jump height 2.63, jump power 3.22, RSI 0.07. At all test parameters, the results in the final test were better than in the final test, the highest progress was recorded at the jump height of 129.0% for the left foot and 159.5% for the right foot. In this test the Cohen’s values (d) were between 0.01 and 2.76 and the effect size was over 0.08 wide, for all parameters, except for the contact time for the left/right foot and the RSI for the left foot where the size of the effect was small and medium. All the analyzed parameters registered statistically significant results, with the exception of the parameters ‘contact time’ and ‘RSI’ for the executions on the left leg.

The results recorded in the 60 sec vertical jump test (Table 4) were statistically significant for *p* < 0.05, except for flight time where p was 0.731. By analyzing the results, we highlight the following differences: contact time 0.063, flight time 0.83, jump height 16.51, jump power 22.82, RSI 0.89. At all test parameters the results in the final test were better than in the initial test, the highest progress was recorded at the jump height 324.5% and RSI 292.7%, and the lowest progress at flight time 2.1%. Most of the parameters analyzed in this test recorded a large effect size of over 0.08, except for the flight time of only 0.11, having a small effect.

The analysis of the data presented in Table 5 allows us to highlight the fact that all the results were statistically significant at all parameters of the BFS vertical jump test, except for the contact time, which was p 0.494. The highest progress was in the RSI parameters 97.1%, and jump height 4.4%, and the lowest in contact time of 5.3%. Comparing the average of the differences recorded between the two initial and final tests, the following relevant results are highlighted: the contact time improved by 0.041, the flight time was better by 0.089, the jump height was better at the final test by 7.94, the power of the jump improved by 5.30 and RSI.2. At all test parameters, Cohen’s (d) values ranged from 0.23 to 0.96, reflecting a small effect size for contact time, an average effect size for the bounce height, and a large effect size for the contact time, which widens the parameters specific to the BFS vertical jump test.

## 4. Discussion

The study aimed at optimizing the development of the explosive force of the players—women’s elite football-tennis—by implementing a complex physical training program, which was varied and individualized according to the sports and fasting of the players. The results of our study can be considered to contribute to the expansion of knowledge on the impact of physical training on the deadly and technical performance in women’s football-tennis. All tests of the experiment were monitored with the help of Opto Jump, and significant progress was made in the final test compared to the initial one, which demonstrates the efficiency of the physical training program implemented for the development of explosive force, with an impact on the sports performance of elite players.

The most relevant results were obtained for the left leg, regarding the improvement of the explosive force of the lower limbs materialized in the jump height parameter, which occurred during the test of five vertical jumps on one leg on the spot, and for the right leg in the tests of five back-and-forth jumps and five left/right side jumps. The most significant advances in the study were in the tests, in descending order of their weight: vertical jumps on both legs 60 s; five back-and-forth jumps and five left/right side jumps, five vertical jumps on one leg from the spot, and BFS vertical jumps. The game of football-tennis takes place on a small area, it is an ambidextrous game, and for elite players it is necessary to have equal coordination, strength, speed, and power of execution on both lower limbs. Practicing the high-performance football-tennis game requires from the players a special technical level in performing specific game actions with both the right foot and the left foot. The arguments stated above led us to apply the tests for the lower limbs, regardless of the dominant leg. To optimize training stimuli when the player is with or without the ball, providing data on the specific skills of the game of football-tennis and to maximize the motor actions related to the game, as well as the intensity of training, can be beneficial. The specific physical evaluations are made in correlation with the general physical training of the player according to their annual plan (macrocycle, mesocycle, and microcycle) of the stages of preparation and competition (preparatory, pre-competitive, competitive, and transition).

The specialized literature highlights very few studies on football-tennis, and the many identified studies focus mainly on the management of the game and the technical training of football-tennis players [53,54]. We consider the results of our study to complete the conclusions of recent studies conducted on 12 football players [55], respectively, and on 40 football players [56], over a period of 8 months of endurance training combined with plyometric exercises/strength training program, which showed significant improvements in muscle strength and jump ability [57,58]. The level of explosive force can contribute to improving performance and sporting success. In this sense, a study conducted on 306 senior male soccer players highlighted the correlation between jumping height (countermovement jump and standing jump) and team performance [59]. Other studies show positive correlations between speed, agility, and performance at various jump tests to highlight the explosive force of the lower limbs [60,61,62]. Studies conducted on athletes of different levels of training have shown the positive correlation between the level of development of the other motor qualities and the explosive force [63,64,65,66,67,68].

Scientific studies that address the issue of the game of football-tennis are much less compared to those aimed at football. A series of studies approach the complexity of sports training in football, with the possibility of adaptation for football-tennis aiming at the correlation between the vastness of football players and their anthropometric characteristics [69]; as well as from the perspective of some determining factors such as the location of the match, the type of turf, and the result of the match [70]. Some studies about the impact of the development of the strength of football players with the possibility of extrapolation to football-tennis have focused on strength training models on strength and muscle strength in performance footballers, and on complex training models aimed at optimizing explosive skills in correlation with speed and coordination [70,71,72]. The approach of sports technique is a permanent concern of experts whose studies have focused mainly on identifying the impact of developing maximum hitting speed in relation to the use of different types of ball [73,74,75]; as well as the correlation between technical level and different types of training [76,77,78].

The identified limitations of the study were the relatively small number of athletes included in the study; limiting the study only to the female sample; analyzing only the explosive force evaluation tests without correlating with anthropometric parameters, technical level, or other essential motor qualities in training of football-tennis athletes. The strong points of the study were the study’s inclusion of the female athletes who are part of the Romanian national team and medalists at the world and European championships; the use of the Opto Jump equipment and software to evaluate the explosive force on the hind limbs; the large number of jump tests to evaluate the explosive force in regime of speed, resistance, and dynamic balance; the exercise program implemented, which comprised plyometric exercises, jumping exercises, technical exercises with acceptance on hanging on one leg or both legs, passes from jumping, hitting the ball from the jump, etc.; and that the program was individualized according to the sports features of the subjects included in the study.

## 5. Conclusions

The explosive force can be optimized through a physical training program that must be individualized and adapted to the level of performance of football-tennis athletes. The explosive force has an important impact in improving the physical fitness of the football-tennis players with a major role in achieving the technical-tactical mastery of sports. Implementing a varied program of exercises, such as plyometric exercises, jumping exercises, technical exercises with acceptance on hanging on one leg or both legs, passes without jumping, hitting the ball from the jump, etc., determines the development of the explosive force in regime of speed, resistance, and dynamic balance, all essential components of sportsmanship at the level of the world and European elite echelon.

Football-tennis contributes to the education and development of specific motor qualities, implicitly to the optimization of physical and technical-tactical training. The permanent and dynamic adaptation of the training program are essential premises for sports success at the level of elite performances. Physical training in football-tennis must be based on the capacity for effort, which needs to be permanently improved, as well as the technical and tactical training required to be able to easily conclude a point as quickly and efficiently as possible.

## Figures and Tables

**Table 1:** Statistical description of the test of five vertical jumps on the spot, on the left/right leg.

Test	Parameters	Leg	Ti—X ± SD	Tf—X ± SD	∆XTI-TF	95% C.I. Lower; Upper	PG %	t	*p*	d
Five vertical jumps on the spot, on the left/ right leg	Contact time	Left	0.28 ± 0.04	0.31 ± 0.03	0.028	−0.01; 0.07	9.81	1.46	0.17	0.46
		Right	0.29 ± 0.03	0.32 ± 0.03	0.032	0.01; 0.05	11.14	3.65	0.00	1.15
	Flight time	Left	0.26 ± 0.06	0.31 ± 0.09	0.054	0.01; 0.12	12,16	2.32	0.01	0.76
		Right	0.25 ± 0.06	0.32 ± 0.09	0.073	0.07; 0.13	29.23	2.52	0.03	0.80
	The height of the jump	Left	6.47 ± 4.53	12.91 ± 7.03	6.44	1.90; 1.98	99.57	3.21	0.01	1.01
		Right	8.01 ± 4.26	13.53 ± 6.45	5.52	1.25; 9.79	68.92	2.92	0.01	0.92
	The power of the jump	Left	1.18 ± 5.13	16.45 ± 7.39	6.27	1.22; 11.31	61.64	2.81	0.02	0.89
		Right	11.97 ± 5.36	17.41 ± 7.14	5.44	0.64; 1.25	45.52	2.56	0.03	0.81
	RSI	Left	0.21 ± 0.17	0.46 ± 0.26	0.26	0.09; 0.39	113.14	3.67	0.00	1.16
		Right	0.29 ± 0.19	0.49 ± 0.26	0.20	0.04; 0.37	69.34	2.75	0.02	0.87

Ti—initial test, Tf—final test, X—arithmetic mean, SD—standard deviation, t—Student test, (∆XTI-TF—average differences between final test and initial test, 95% C.I—interval of confidence lower and upper, PG %—percent of progress, d—effect size, RSI—Reactive Strength Index.

**Table 2:** Statistical description of the test of five back-and-forth jumps on the left/right leg.

Test	Parameters	Leg	Ti—X ± SD	Tf—X ± SD	∆XTI-TF	95% C.I. Lower; Upper	PG %	t	*p*	d
Five back-and-forth jumps on the left/ right leg	Contact time	Left	0.30 ± 0.02	0.35 ± 0.06	0.056	0.00; 0.11	18.31	2.29	0.04	0.72
		Right	0.28 ± 0.02	0.56 ± 0.40	0.282	−0.01; 0.58	99.73	2.15	0.04	0.68
	Flight time	Left	0.17 ± 0.07	0.28 ± 0.07	0.109	0.03; 0.18	62.12	3.26	0.01	1.03
		Right	0.17 ± 0.05	0.41 ± 0.26	0.237	0.02; 0.44	135.11	2.54	0.03	0.80
	Height of jump	Left	4.90 ± 2.98	1.63 ± 5.25	5.73	2.41; 9.05	116.97	3.91	0.00	1.24
		Right	4.09 ± 2.55	11.23 ± 4.63	7.14	3.65; 1.63	174.62	4.63	0.00	1.46
	Power of jump	Left	7.85 ± 2.39	15.83 ± 9.75	7.98	0.64; 15.31	101.56	2.46	0.03	0.78
		Right	7.46 ± 2.91	15.44 ± 5.93	7.98	3.66; 12.30	107.02	4.18	0.00	1.32
	RSI	Left	0.14 ± 0.06	0.47 ± 0.45	0.33	0.00; 0.67	247.15	2.25	0.05	0.71
		Right	0.17 ± 0.08	0.47 ± 0.24	0.30	0.12; 0.49	184.32	3.80	0.00	1.20

Ti—initial test, Tf—final test, X—arithmetic mean, SD—standard deviation, t—Student test, (∆XTI-TF—average differences between final test and initial test, 95% C.I—interval of confidence lower and upper, PG %—percent of progress, d—effect size, RSI—Reactive Strength Index.

**Table 3:** Statistical description of the test of five left/right side jumps on the left/right leg.

Test	Parameters	Leg	Ti—X ± SD	Tf—X ± SD	∆XTI-TF	95% C.I. Lower; Upper	PG %	t	*p*	d
Five left/right side jumps on the left/right leg	Contact time	Left	0.40 ± 0.17	0.343 ± 0.06	−0.057	−0.207; 0.093	14.38	0.86	0.41	0.27
		Right	0.29 ± 0.04	0.359 ± 0.05	0.066	0.019; 0.112	22.32	3.18	0.01	0.01
	Flight time	Left	0.17 ± 0.08	0.28 ± 0.05	0.109	0.053; 0.166	63.71	4.38	0.00	1.39
		Right	0.19 ± 0.07	0.29 ± 0.05	0.098	0.062; 0.135	5.82	6.16	0.00	1.95
	Height of jump	Left	4.62 ± 3.89	1.58 ± 4.23	5.96	3.34; 8.58	129.04	5.15	0.00	1.63
		Right	5.09 ± 4.45	13.21 ± 3.96	8.12	5.77; 1.47	159.52	7.82	0.00	2.47
	Power of jump	Left	9.79 ± 3.85	13.33 ± 4.17	3.54	1.11; 5.97	36.1	3.29	0.00	1.04
		Right	8.65 ± 5.07	16.55 ± 4.78	7.90	5.84; 9.94	91.2	8.71	0.00	2.76
	RSI	Left	0.23 ± 0.26	0.34 ± 0.16	0.11	−0.05; 0.27	49.14	1.56	0.15	0.49
		Right	0.19 ± 0.16	0.37 ± 0.15	0.18	0.10; 0.27	97.86	4.77	0.00	1.51

Ti—initial test, Tf—final test, X—arithmetic mean, SD—standard deviation, t—Student test, (∆XTI-TF—average differences between final test and initial test, 95% C.I—interval of confidence lower and upper, PG %—percent of progress, d—effect size, RSI—Reactive Strength Index.

**Table 4:** Statistical description of the 60 s vertical jump.

Test	Parameters	Ti—X ± SD	Tf—X ± SD	∆XTI-TF	95% C.I. Lower; Upper	PG %	t	*p*	d
60 s vertical jump	Contact time	0.183 ± 0.070	0.246 ± 0.030	0.063	0.014; 0.112	34.42	2.91	0.01	0.92
	Flight time	0.411 ± 0.698	0.494 ± 0.199	0.083	−0.445; 0.610	2.14	0.36	0.73	0.11
	Height of jump	5.09 ± 3.84	21.60 ± 1.04	16.51	9.46; 23.56	324.52	5.30	0.00	1.67
	Power of jump	1.67 ± 7.99	33.49 ± 13.87	22.82	13.33; 32.30	213.84	5.44	0.00	1.72
	RSI	0.30 ± 0.26	1.19 ± 0.64	0.89	0.53; 1.24	292.78	5.65	0.00	1.79

Ti—initial test, Tf—final test, X—arithmetic mean, SD—standard deviation, t—Student test, (∆XTI-TF—average differences between final test and initial test, 95% C.I—interval of confidence lower and upper, PG %—percent of progress, d—effect size, RSI—Reactive Strength Index.

**Table 5:** Statistical description of the BFS vertical jump test.

Test	Parameters	Ti—X ± SD	Tf—X ± SD	∆XTI-TF	95% C.I. Lower; Upper	PG %	t	*p*	d
BFS vertical jumps	Contact time	0.779 ± 0.164	0.738 ± 0.097	−0.041	−0.172; 0.089	5.37	0.71	0.49	0.23
	Flight time	0.393 ± 0.080	0.482 ± 0.058	0.089	0.013; 0.164	22.51	2.64	0.02	0.84
	Height of jump	19.65 ± 7.34	27.59 ± 6.21	7.94	0.56; 15.32	4.44	2.43	0.03	0.77
	Power of jump	14.51 ± 3.83	19.81 ± 3.90	5.30	0.80; 9.81	36.52	2.66	0.02	0.84
	RSI	0.21 ± 0.15	0.41 ± 0.14	0.20	0.05; 0.36	97.14	3.05	0.01	0.96

Ti—initial test, Tf—final test, X—arithmetic mean, SD—standard deviation, t—Student test, (∆XTI-TF—average differences between final test and initial test, 95% C.I—interval of confidence lower and upper, PG %—percent of progress, d—effect size, RSI—Reactive Strength Index.
